# A tunable artificial circadian clock in clock-defective mice

**DOI:** 10.1038/ncomms9587

**Published:** 2015-11-30

**Authors:** Matthew D'Alessandro, Stephen Beesley, Jae Kyoung Kim, Rongmin Chen, Estela Abich, Wayne Cheng, Paul Yi, Joseph S. Takahashi, Choogon Lee

**Affiliations:** 1 Department of Biomedical Sciences, Program in Neuroscience, College of Medicine, Florida State University, 1115 West Call Street, Tallahassee, Florida 32306, USA; 2 Mathematical Biosciences Institute, The Ohio State University, 1735 Neil Avenue, Columbus, Ohio 43210, USA; 3 Department of Mathematical Sciences, Korea Advanced Institute of Science and Technology, Daejeon 305-701, Korea; 4 Howard Hughes Medical Institute, Department of Neuroscience, University of Texas Southwestern Medical Center, Dallas, Texas 75390, USA

## Abstract

Self-sustaining oscillations are essential for diverse physiological functions such as the cell cycle, insulin secretion and circadian rhythms. Synthetic oscillators using biochemical feedback circuits have been generated in cell culture. These synthetic systems provide important insight into design principles for biological oscillators, but have limited similarity to physiological pathways. Here we report the generation of an artificial, mammalian circadian clock *in vivo*, capable of generating robust, tunable circadian rhythms. In mice deficient in *Per1* and *Per2* genes (thus lacking circadian rhythms), we artificially generate PER2 rhythms and restore circadian sleep/wake cycles with an inducible *Per2* transgene. Our artificial clock is tunable as the period and phase of the rhythms can be modulated predictably. This feature, and other design principles of our work, might enhance the study and treatment of circadian dysfunction and broader aspects of physiology involving biological oscillators.

## Abstract (web summary)

Circadian rhythms are central to health and disease and there is renewed interest in chronotherapy. Here, the authors present a mouse with an artificial circadian clock that can be pharmacologically tuned, providing a tool for future studies of circadian biology and therapy.

## Graphical abstract

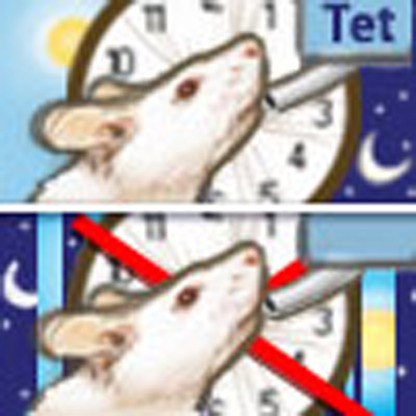

In mammals, important daily activities such as sleep/wake cycles and metabolic homeostasis are governed by the circadian clock, a genetically determined, endogenous timekeeper that can adjust to environmental cues, such as the day/night cycle^1^,^2^. The clock operates cell autonomously and is driven and self-sustained by a negative-feedback loop consisting of positive and negative transcriptional control. A negative-feedback loop is fundamental to the circadian oscillator in most organisms^3^,^4^,^5^,^6^, as diverse as *Cyanobacteria*, *Neurospora*, *Drosophila* and mammals^7^. In mammalian clock cells, CLOCK (or NPAS2) and BMAL1 are the positive elements, activating transcription of many downstream genes including the negative elements, *Period* (*Per*) and *Cryptochrome* (*Cry*), whose products form an inhibitory complex^2^,^8^. Pioneering *Drosophila* studies led by Hardin *et al.*^9^ were the first to support the model that circadian rhythms can be generated by a transcriptional negative-feedback loop. Guided by these findings and computational design principles^3^,^10^, mathematical modelling confirmed that self-sustaining oscillations can be generated by a negative-feedback loop constructed with built-in delayed feedback inhibition^4^,^5^,^6^. The current working model for the mammalian clock is much more complicated than the original negative-feedback model, with several interlocked negative- and positive-feedback loops^2^,^11^, but the principle remains the same. A core feedback-loop-driving oscillations in the PER:CRY inhibitor complex is essential for rhythmicity, while other feedback loops that drive oscillations in the CLOCK:BMAL1 activator complex may contribute to robust rhythmicity^12^,^13^. In the inhibitor complex, PER is the stoichiometrically rate-limiting component; its phosphorylation kinetics, balanced by kinases and phosphatases, functions as a circadian timer, determining period and phase^14^,^15^.

There is considerable interest in engineering genetic circuits to simulate diverse biochemical oscillators, such as the cell cycle, the circadian clock, and the insulin secretion pathway^3^,^16^,^17^,^18^,^19^,^20^. Results have demonstrated proof of principle that self-sustaining, synthetic oscillators could be constructed using computational algorithms and experimental parameters. However, none of these synthetic oscillators can be practically tied to *in vivo* circadian physiology as they employ artificial reporters regulated by heterologous genetic networks.

To provide design principles for building a synthetic circadian clock that can be functional *in vivo* and control natural circadian physiology, such as wake/sleep rhythms, we generate an artificial genetic circuit. This tunable *Per2* oscillator is generated *in vivo* by crossing tetracycline (Tet)-controlled transgenic *Per2* mice (also responsive to doxycycline (Dox))^21^ with *Per1/2* double knockout mice^22^. We show that exogenous (Tet driven or Dox driven) oscillations of transgenic *Per2 (tPer2)* can restore molecular and behavioural rhythms to *Per*-knockout mice. We also show that transgenic *Per1* has the same potential for an artificial clock. Although we use the PER2 protein to interface with endogenous clock output pathways, this is not a simple rescue experiment because the endogenous clock feedback loop is no longer the main timekeeping mechanism in these mice; it is supplanted by an artificial mechanism driven by Tet or Dox. Our work shows that many of the endogenous genetic circuits in the current clock model are dispensable, and further study of our artificial clock may help to distinguish critical mechanisms of clock regulation. Furthermore, the design principles of our approach may be applied to engineer other artificial oscillators for the study of other physiological processes.

## Results

### A tunable circadian oscillator generated by transgenic PER

**Figure 1:**
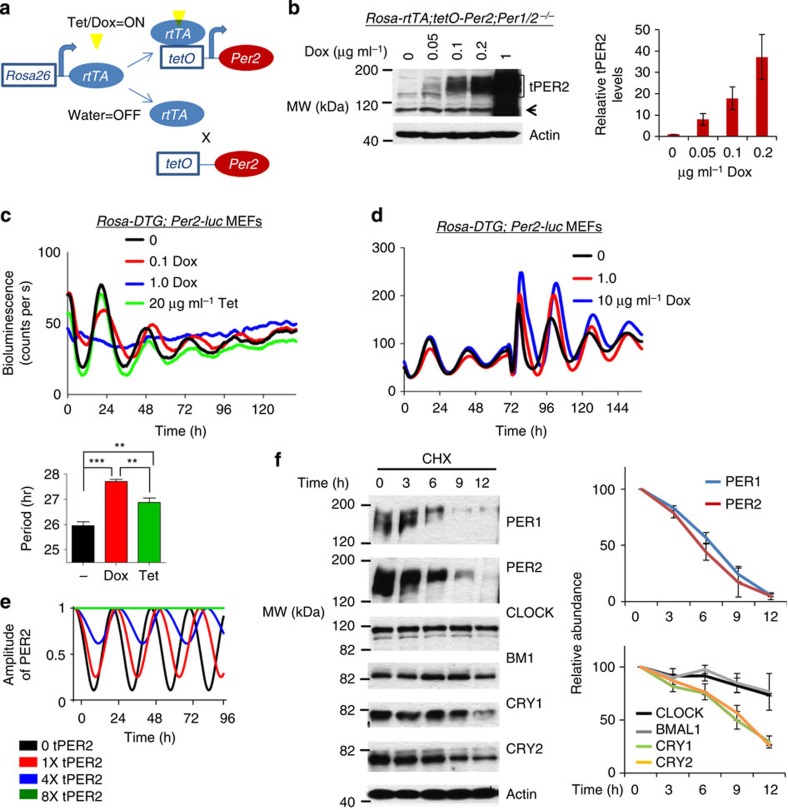
A tunable artificial oscillator generated by synthetic PER oscillations. (**a**) Conditional transgenic *Per2 (tPer2)* expression by Tet-ON. *tPer2* transcription is activated by a universal rtTA, *Rosa26-rtTA*, only when Tet or Dox is present. (**b**) Expression of tPER2 in a dose-responsive manner. tPER2 expression was measured at different Dox concentrations in Rosa-DTG; *Per1/2* double mutant (*Per1/2*^*−/−*^) MEFs. There were low levels of basal *tPer2* expression in the absence of drugs (Supplementary Fig. 1). The arrowhead indicates a non-specific band. Values are mean+/−s.e.m., *n*=3. (**c**) Period lengthening in a PER2 dose-responsive manner. The period was measured in the presence of different amounts of Dox and Tet. See also Supplementary Fig. 2a. Period was significantly lengthened by Dox (*P*<0.001) and Tet (*P*<0.01). Period with Dox treatment was longer than that with Tet (*P*<0.01). *P<0.05, **P<0.01, ***P<0.001 (unpaired, two-tailed *t*-test). Values are mean+/−s.e.m., *n*=4. (**d**) Phase shifts by a 2-h pulse of Dox. Phase shifts were measured after baselines were measured for ∼2.5 days before the pulse. (**e**) Simulation of a similar experiment as in **c** using a mathematical model (Supplementary Fig. 3). tPER2 levels changed from zero- to eightfold higher than those of endogenous PER2. Note that period and amplitude are altered in a dose-responsive manner as in **c**. According to our model, period is lengthened as PER2 levels increase because it takes more time to degrade the rate-limiting inhibitor in the nucleus and relieve feedback inhibition for the next cycle. (**f**) Half-lives of major clock proteins. Six major clock proteins were measured at indicated times after WT MEFs were treated with cycloheximide (CHX). Values are mean+/−s.e.m.. *n*=3 for CLOCK, CRY1 and 2, *n*=4 for the others.

As a first step, we tested the Tet-controlled synthetic oscillator in cultured mouse embryonic fibroblasts (MEFs) and validated that the cellular system is tunable in a dose-responsive manner (Fig. 1). We generated double transgenic (DTG) mice with the *tetO-Per2* transgene (*tPer2*) and a ubiquitiously expressed reverse Tet-controlled TA driver, *Rosa26-rtTA* (Tet-ON) in the double knockout (DKO) *Per1/2*^*−/−*^ background (Fig. 1a). When MEFs from this Rosa-DTG/DKO mouse were subjected to varying doses of Dox, tPER2 was expressed in a dose-dependent manner (Fig. 1b). Dox between 0.05 and 0.1 μg ml^−1^ induced *tPer2* expression comparable to endogenous levels of PER2 when measured in Rosa-DTG; *Per2-luc* MEFs (*Per2*^*Per2-luc/+*^; Supplementary Fig. 1). Period and phase in these cells were altered by continuous treatment of Dox and 2-h pulses of Dox, respectively, in a dose-responsive manner (Fig. 1c,d and Supplementary Fig. 2a). Circadian rhythms could not be sustained if *tPer2* expression was constitutively too high, as we have shown previously (Fig. 1c)^21^.

Circadian rhythms are lengthened if the ratio of PER to CK1δ/ɛ is increased by deleting alleles of *CK1*δ*/*ɛ or disrupting CK1δ/ɛ with a dominant-negative CK1δ or ɛ (refs 14, 23, 24). The same relationship was maintained in our inducible *tPer2* cells. When period was lengthened by tPER2 overexpression in Rosa-DTG; *Per2-luc* MEFs, this was rescued by restoring the stoichiometry between PER and CK1δ/ɛ through transgenic expression of CK1δ, demonstrating that our inducible *tPer2* system is also controlled by the same posttranscriptional mechanisms as the endogenous one (Supplementary Fig. 2b).

Wild-type (WT) MEFs with a Tet-controlled *Per1* transgene exhibited similar changes in period and amplitude in a dose-responsive manner (Supplementary Fig. 2c), indicating that *Per1* has the same potential as *Per2* for an artificial oscillator. Tetracycline was less potent than Dox in activating tPER2 and altering period in MEFs (Fig. 1c, Supplementary Fig. 1). Similar results could be generated from our mathematical model (Supplementary Fig. 3): period and amplitude changed in a dose-dependent manner when constitutive tPER2 levels gradually increased in a *Per* WT background (Fig. 1e). In both *in vitro* and *in silico*, circadian rhythms could not be generated from DTG/DKO cells by continuous Dox or Tet treatment, indicating that rhythmic *tPer2* transcription is necessary for generating circadian rhythms (Supplementary Fig. 4a,b). Further, when stability of endogenous clock proteins was compared, PER1 and 2 had the shortest half-lives (Fig. 1f), making them the best candidates to generate robust oscillations^19^.

### Robust rhythms generated by the artificial oscillator

We next tested if the Tet-controlled *tPer2* system can generate rhythms *in vivo*. We generated *Scg2-tTA-DTG/DKO* mice, where *tPer2* expression occurs in neurons, including the SCN (suprachiasmatic nuclei, site of the master clock in mammals) in the absence of Tet (Tet-OFF). We could not use the Rosa-DTG system because *Rosa26* is not expressed in the SCN. Like the Rosa-DTG system, this system takes advantage of the existing endogenous circadian genetic network: we did not have to supply a transgenic activator for circadian output or reporter genes, because the tPER2 would regulate endogenous CLOCK and BMAL1 transcriptional activators; and the necessary time-delay mechanisms for PER2 accumulation^3^, and thus for PER:CRY accumulation and feedback inhibition of CLOCK:BMAL1, should be intact because *tPer2* has the same coding sequence and 3′-UTR as WT *Per2.* It has been shown that the time delay in PER accumulation in the cytoplasm mainly occurs by regulation at the translational level through the 3′-UTR of *Per2* and at posttranslational levels^25^,^26^,^27^. *In vivo*, we expected that oscillations of *tPer2* would be self-sustained once circadian behaviour is initiated because concentrations of Tet in the blood would oscillate due to circadian drinking behaviour: low and high Tet during subjective day and night, respectively. We chose Tet over Dox because it has a shorter half-life^28^ and therefore would oscillate more robustly in the blood, which should produce stronger amplitudes in *tPer2* oscillations and behavioural rhythms. This was predicted by our mathematical model and confirmed by comparison experiments between Tet and Dox (Supplementary Fig. 4b–d).

**Figure 2:**
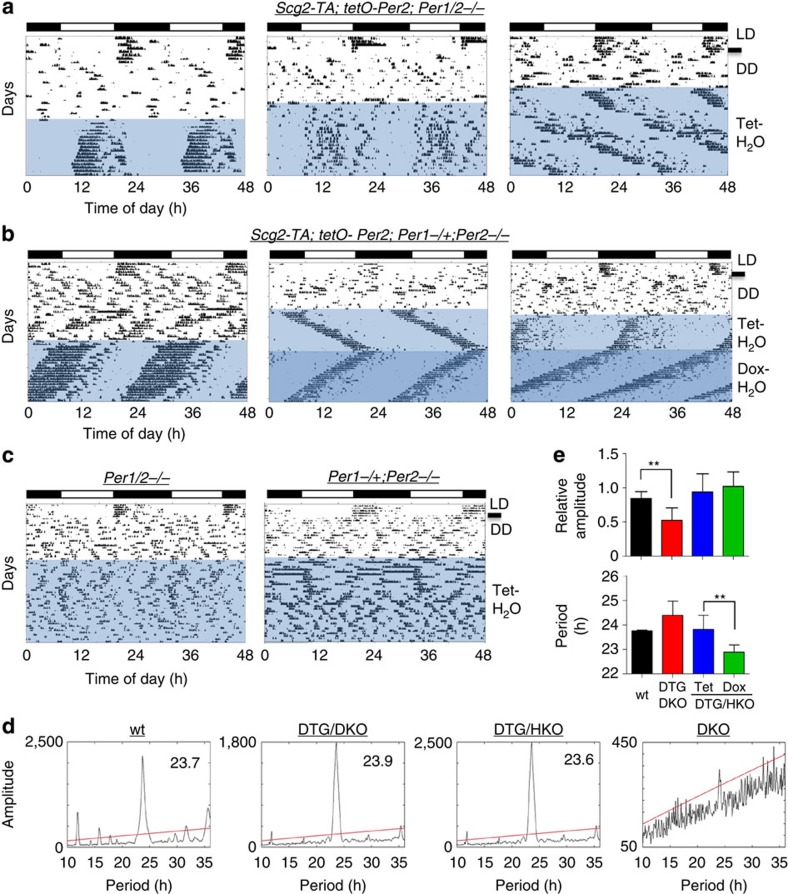
Synthetic *Per2* oscillations can generate robust rhythms in clock-deficient mice. (**a**) Representative actograms from Scg2-DTG/DKO mice on Tet *ad libitum*. White and blue areas represent regular and Tet water, respectively. (**b**) Representative actograms from Scg2-DTG/HKO mice on Tet or Dox *ad libitum*. Light and dark blue represent Tet and Dox treatment, respectively. (**c**) Representative actograms from DKO and HKO control mice. (**d**) Representative χ^2^- graphs showing the period and amplitude of rhythms for four different genotypes. The actogram for the WT mouse is shown in Supplementary Fig. 5. (**e**) Quantitative analysis of circadian parameters. *n*=8, 7, 7, 6 for WT, DTG/DKO, DTG/HKO-Tet and DTG/HKO-Dox, respectively. Values are mean+/−s.e.m. *P<0.05, **P<0.01, ***P<0.001 (unpaired, two-tailed *t*-test).

**Figure 3:**
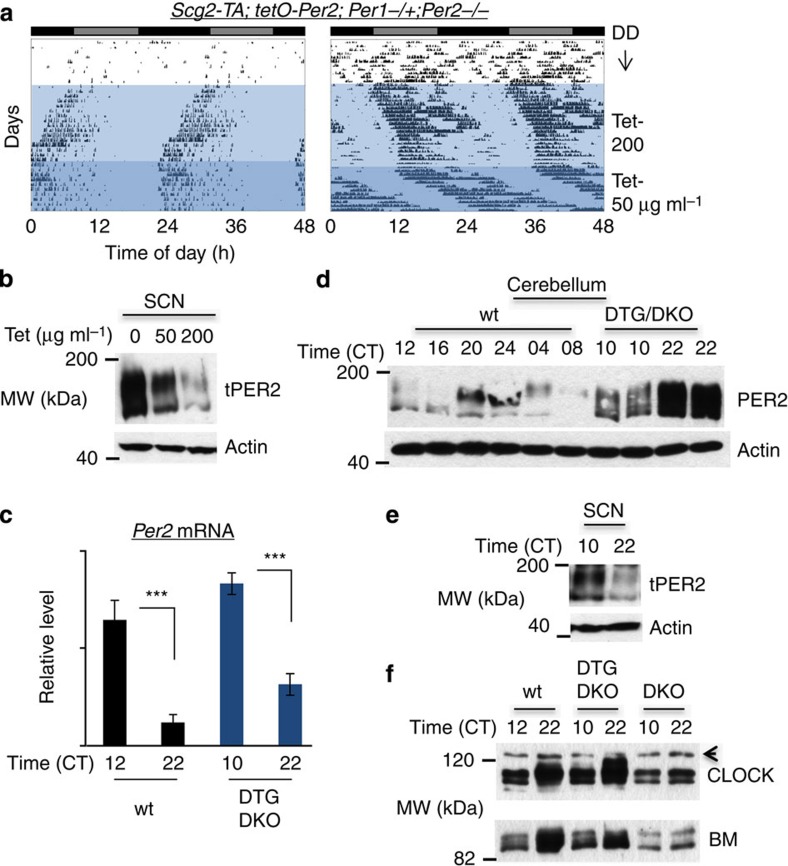
The artificial clock is integrated into the endogenous clock network. (**a**) Period of circadian rhythms changed in a dose-dependent manner as in cultured cells. Representative actograms from Scg2-DTG/HKO mice on regular water, 200 μg ml^−1^ Tet and then 50 μg ml^−1^ Tet *ad libitum*. Period at 50 μg ml^−1^ Tet was longer than at 200 μg ml^−1^ Tet by 49.2±14.4 min (*n*=4). (**b**) Tet dose-dependent expression of tPER2 in SCN. SCN were pooled from three DTG/DKO mice at each Tet dose at ZT 22. (**c**,**d**) Oscillations of *tPer2* mRNA and protein in cerebellum. Cerebellum from individual mice was used to measure *tPer2* mRNA and protein because a single SCN punch does not provide enough protein for PER immunoblots and mRNA for QT–PCR^27^. Tissue was harvested from rhythmic mice based on circadian times when they were robustly rhythmic. WT *Per2* mRNA at peak and trough levels in cerebellum were compared with those of *tPer2* in DTG/DKO (**c**). Values are mean±s.e.m., *n*=4. *P<0.05, **P<0.01, ***P<0.001 (unpaired, two-tailed *t*-test). Oscillations of tPER2 in cerebellum are shown in (**d**). Two different mice are shown for tPER2 at CT10 and 22. (**e**) tPER2 has a different phase in SCN relative to that in cerebellum. SCN were collected and pooled from three DTG/DKO mice at CT 10 and 22. Note that tPER2 is more abundant at CT10 than at CT22, unlike in cerebellum. (**f**) Rhythmic CLOCK and BMAL1 in DTG/DKO. The same tissues as in **c**,**d** were used. The arrow indicates a non-specific band. Note that CLOCK is hyperphosphorylated when PER2 is abundant in WT and DTG/DKO mice^21^.

We initiated a circadian cycle in the DTG/DKO mice by changing the water bottles from regular water to Tet water, which should jump start *tPer2* cycles. As shown in Fig. 2, DTG/DKO mice exhibited robust rhythms when they were on Tet *ad libitum* after the initial pulse start (Fig. 2a,d,e). When compared with WT control mice (Supplementary Fig. 5), phase angle was unstable and amplitude was low in these mice. Some of the mice lost rhythmicity gradually (Supplementary Fig. 6a) but others maintained rhythms as long as they were on Tet water. The artificial oscillator also induced rhythms in arrhythmic *Per1*+/−*; Per2−/−* mice (DTG/HKO) and *Per1−/−; Per2*+/− mice (DTG/KOH) given the same treatment (Fig. 2b,d,e, Supplementary Fig. 6b). Amplitude and stability (phase angle) of the rhythms were as robust as those in WT mice. Robustness of the rhythms did not deteriorate over the course of measurement. The induced rhythms in all these mice disappeared almost immediately once Tet water was switched to regular water (Supplementary Fig. 6c). In DTG/HKO mice, the period was shorter when the mice were on Dox than on Tet, probably because Dox is more potent at inhibiting tTA and thus results in lower *tPer2* expression, which is consistent with the results in cultured cells with the Tet-ON system. The same treatment in DKO and HKO control mice did not produce significant rhythmicity (Fig. 2c). As in cultured cells with the Tet-ON system, circadian period and PER expression in the SCN were tunable in a dose-responsive manner (Fig. 3a,b). When cerebellum was harvested based on circadian times while DTG/DKO mice were robustly rhythmic, *tPer2* mRNA and protein levels were rhythmic as predicted (Fig. 3c,d). In addition, tPER2 phase was delayed relative to that of mRNA levels in DTG/DKO mice, demonstrating that time-delay mechanisms in tPER2 accumulation are intact in these mice (compare the mRNA and protein rhythms in Fig. 3c,d). Interestingly, the phase of tPER2 is apparently different between SCN and cerebellum (Fig. 3d,e), suggesting that the phase advance in the SCN relative to other peripheral tissues may be generated at both transcriptional and posttranscriptional levels^29^. Rhythms were generated by the artificial oscillator in DTG/DKO and DTG/HKO mice because the oscillator could be integrated into the endogenous molecular network, producing circadian rhythms in other clock genes and downstream genes; CLOCK and BMAL1 exhibited similar oscillations in abundance and phosphorylation in DTG/DKO as in WT mice, whereas these proteins were constitutive in DKO mice (Fig. 3f).

**Figure 4:**
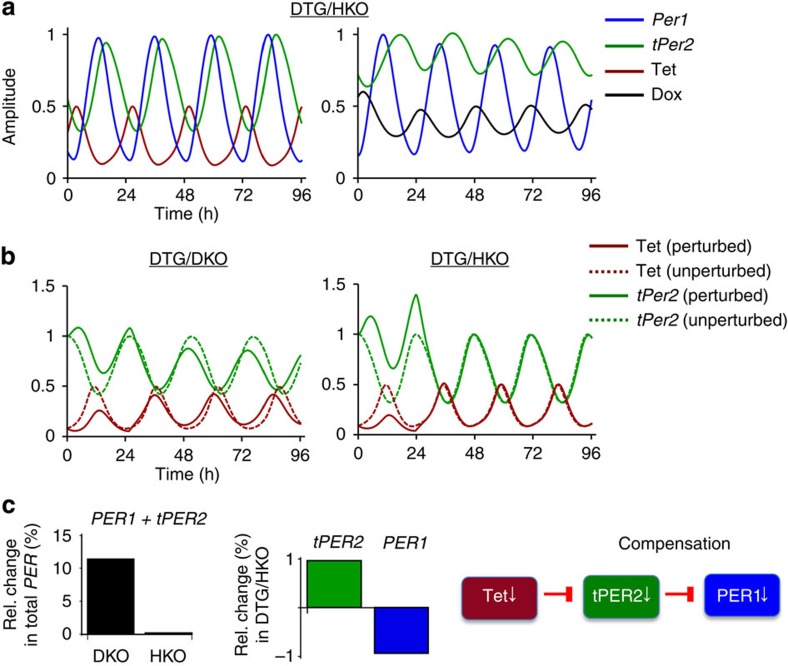
*Per1* and *tPer2* alleles synergistically contribute to robustness of rhythms. (**a**) Synergistic oscillations between *Per1* and *tPer2*. Relative amplitudes for endogenous *Per1* and *tPer2* rhythms are depicted in the models. Note that endogenous *Per1* and *tPer2* rhythms are synchronous and reinforce each other. *Ad libitum* Dox can generate robust *tPer2* rhythms in DTG/HKO mice due to endogenous *Per1* oscillations. (**b**) Resistance of the DTG/HKO oscillator to temporal changes in daily drinking behaviour. Stability of *tPer2* phase was measured after the water consumption had been reduced to a third of the previous daily level for one day in DTG/DKO and DTG/HKO mice. (**c**) Stability of DTG/HKO rhythms is achieved through feedback regulation between endogenous *Per1* and *tPer2*. Increased *tPer2* due to lower consumption of regular water is balanced by decreased *Per1* levels which are mediated by increased feedback from higher levels of tPER2. This would result in insignificant changes in total *Per* levels and rhythms. Note that *tPer2* phase is shifted by the perturbation in DTG/DKO mice, but not in DTG/HKO mice due to the feedback compensation.

Rhythms in the DTG/HKO mice were more robust than in DTG/DKO mice. Our mathematical model predicted that this is because the DTG/HKO oscillator is resistant to variability in daily drinking behaviour through a compensatory mechanism involving endogenous *Per1* (Fig. 4). Temporal up- or downregulation of *tPer2* levels would feedback-regulate endogenous *Per1* levels in the opposite direction, which should correct the temporal perturbation in total *Per* levels and rhythms. WT-like robustness in DTG/HKO mice suggests that the circadian oscillator has built-in mechanisms to absorb perturbations in the system that are more extreme than normal physiological variability.

### Tunable rhythms generated by the artificial oscillator

**Figure 5:**
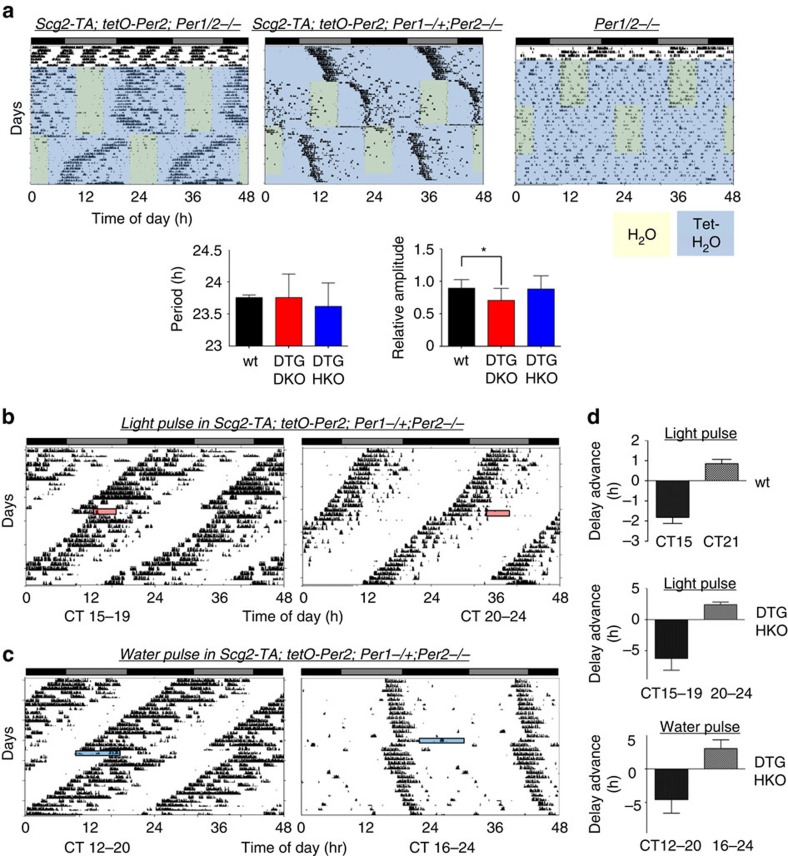
Circadian phase is tunable by shifting or transiently perturbing *tPer2* rhythms. (**a**) Representative actograms of DTG/DKO, DTG/HKO and DKO mice on different schedules of regular water/Tet water entrainment. Tet water treatment is represented by blue shaded areas, and 6-h intervals of regular water by yellow shaded areas. The white area represents continuous regular water treatment. The same treatment did not affect circadian rhythms significantly in WT mice (Supplementary Fig. 7c). Amplitude and period were calculated from the first entrainment. *n*=8, 7, 6 for WT, DTG/DKO and DTG/HKO, respectively. Values are mean±s.e.m. Data for WT period and amplitude were from the same experiments shown in Fig. 2d. *P<0.05, **P<0.01, ***P<0.001 (unpaired, two-tailed *t*-test). (**b**,**c**) Phase shifts by light pulses and by a single regular water treatment. The mice were on Tet *ad libitum* to induce rhythms. Duration and timing of light pulses and water treatment are indicated by red and blue boxes, respectively. (**d**) Quantitative analysis of phase shifts. *n*= 6, 4, 6 for WT mice exposed to light for 1 h, DTG/HKO to light for 4 h, and DTG/HKO to water for 8 h, respectively. DTG/HKO mice required a 4-h light pulse to produce significant phase shifts, probably because a one-hour light pulse does not induce saturating *Per1* induction in the DTG/HKO mice in which only one allele of *Per1/2* can be induced by a light pulse. Values are mean±s.e.m.

The tunability of this oscillator was tested by measuring how rhythms are phase shifted on changing the water-feeding schedule, which would induce *tPer2* at different times of the day in a predictable manner. In both DTG/DKO and DTG/HKO mice, robust rhythms were generated by the scheduled water bottle changes, and rhythms were predictably shifted by changing the water-feeding schedule (Fig. 5a). In all mice, activity onset appeared a few hours after regular water was changed back to Tet water, establishing a strong correlation between phase of induced *tPer2* and onset of activity. Further, when the water schedule was changed by 12 h, there was a clear transitional interval before the phase is locked onto the new schedule. These are reminiscent of entrainment and phase shifts induced by different light:dark (LD) cycles in WT mice, indicating that the artificial oscillator was integrated into the endogenous network.

Using our system, we tested whether temporal perturbation in *Per* expression is indeed the mechanism for phase resetting by light pulses^29^,^30^,^31^ and thus the mechanism underlying jet lag. We hypothesized that DTG/HKO mice can be phase shifted by both light and water pulses because the endogenous *Per1* allele would be induced by light while *tPer2* could be temporally induced by a pulse of regular water. In addition, these mice have stable activity onset that allows us to calculate accurate phase shifts. When these mice were subjected to light pulses at circadian time (CT) 15 and 20, phases were delayed and advanced, respectively, as predicted (Fig. 5b,d). Similarly, rhythms were phase delayed and advanced when these mice were subjected to a water pulse at CT12 and 16, respectively (Fig. 5c,d). The water pulses had to be given earlier to have a similar effect as the light pulses, because there is a lag in *tPer2* induction while residual Tet remains in the blood.

### Generation of a period outside of a normal circadian range

**Figure 6:**
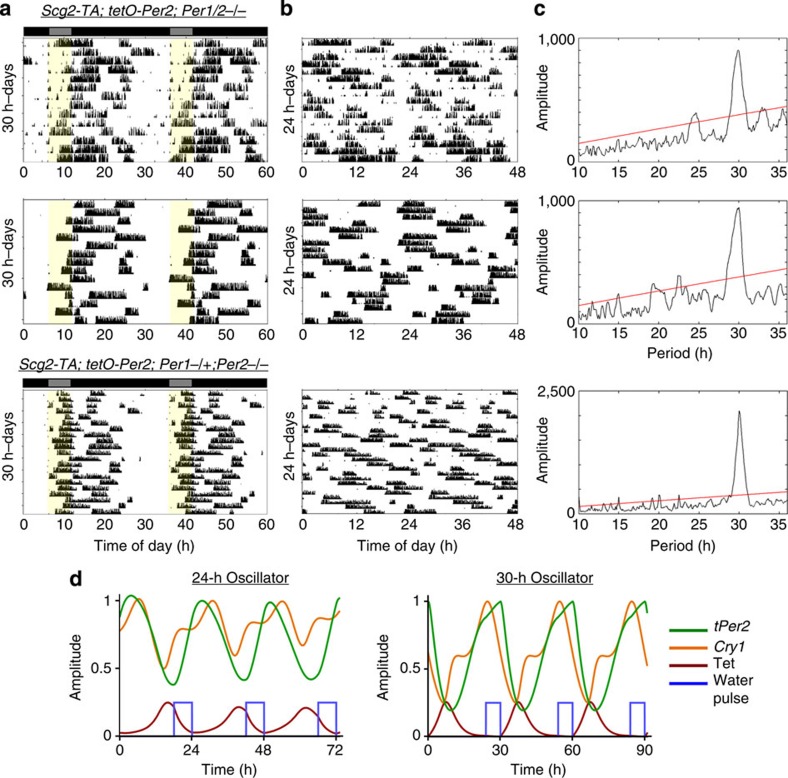
Period tunability of the synthetic oscillator goes beyond a normal range. (**a**) Representative 30-h actograms and χ^2^- graphs from DTG/DKO and DTG/HKO mice. Twenty-four-hour actograms are shown in **b**. The mice were on Tet water continuously except for 6 h in every 30 h, as indicated by yellow shaded areas. Five out of six DTG/DKO and four out of four DTG/HKO mice were rhythmic. Note that the phase of activity onset relative to the water schedule is different from the phase seen during 24-h water entrainment as shown in Fig. 5a. (**c**) Chi-square graphs showing the period and amplitude of rhythms. (**d**) A mathematical model explaining the phase difference in activity onset between 24- and 30-h synthetic cycles. According to our model, oscillations of endogenous clock genes such as *Cry1* are altered during 30-h entrainment, which should produce a different phase in PER:CRY activity and activity onset. Note that endogenous *Cry1* phase relative to synthetic *tPer2* phase is different between the two oscillators. To simulate the 6-h water pulse, we used equation (2). For simplicity of the graphs, scaled *f*(*t*) is described as water-intake rhythms (blue line).

We have thus far shown that exogenously driven *tPer2* oscillations result in robust rhythms and are highly tunable. However, while our interpretation is that the *tPer2* expression system constitutes an artificial oscillator, an alternative explanation may be that the *tPer2* merely jump-starts oscillations of other endogenous clock components such as *Cry*. If the rhythms in the DTG/DKO mice were mainly mediated by endogenous oscillations of other clock components, then the circadian rhythmicity should be constrained to a natural circadian period of close to 24 h. The mammalian clock normally cannot be entrained to extreme cycle lengths such as cycles below 20 and above 28 h (refs 32, 33). For example, if WT mice are placed in a 30-h LD cycle, then they would not be able to entrain to that cycle. When DTG/DKO and DTG/HKO mice were entrained to a 30-h cycle of Tet exposure (6 h regular water to 24 h Tet water), most of the mice (9 out of 10) were robustly rhythmic with a period close to 30 h (Fig. 6a–c). These data strongly indicate that rhythms are directed by artificial oscillations of *tPer2* rather than oscillations of other endogenous clock genes. Phase of activity onset was altered, occurring during the 6-h regular water treatment in the 30-h cycles rather than a few hours after the 6-h water treatment as in the 24-h cycles. Our mathematical model predicted that phase of activity onset (linked to endogenous *Cry1* phase) is different between 24- and 30-h cycles (Fig. 6d). The data demonstrate that rhythms were not generated by simply responding to water bottle changes or tPER2 rhythms. Instead, 30-h rhythmicity could be generated because the endogenous circadian network could be reset to a 30-h cycle by the tPER2 rhythms.

## Discussion

**Figure 7:**
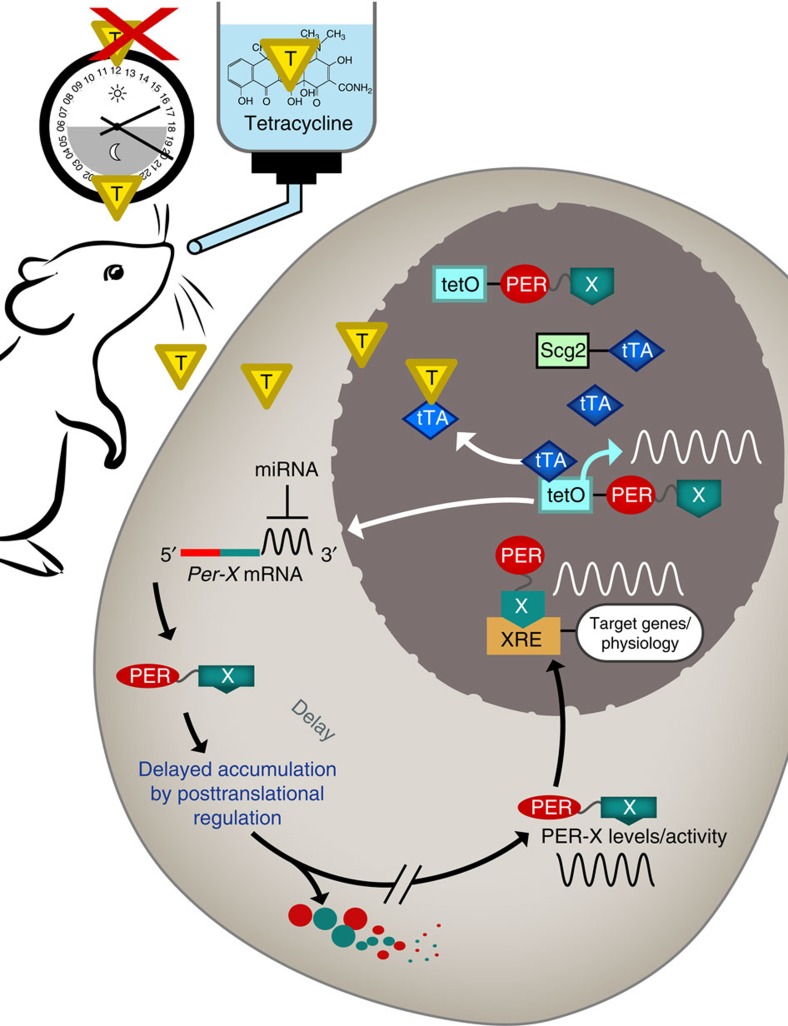
A low-frequency oscillator can be generated using PER protein sequence. Tunable circadian rhythms of a transcription factor X fused to PER2 could be generated using an existing endogenous network. Posttranscriptional mechanisms for *Per2* could generate circadian oscillations of PER2-X fusion protein as in PER2-Luc^45^. In turn, X-controlled pathways would be regulated in a circadian manner *in vivo*.

Here we report a tunable, artificial oscillator using a minimal genetic circuit with only two transgenes, which is disconnected from the endogenous circadian feedback network, yet can direct the endogenous CLOCK:BMAL1-based feedback loop to generate robust rhythms. We predict that PER is the only clock component that can be used in a tunable synthetic oscillator because it is the only proven state variable and has right properties such as a proper half-life and built-in time-delay mechanisms. Although time delay is an essential feature in designing self-sustaining synthetic oscillators^18^,^19^,^20^, no specific mechanisms have been introduced other than inherent time delays during transcription, translation and complex formation, which results in oscillators with very short periods relative to the circadian period. To design a synthetic oscillator based on a negative-feedback loop that can produce a period of ∼24 h, specific time-delay mechanisms must be used to extend the feedback cycle. It has been demonstrated that PER accumulation is delayed at translational and posttranslational levels by unique mechanisms and this is essential for generating a normal circadian period of∼24 h (refs 25, 26, 27). Our water-pulse experiments (Fig. 5b–d) indeed proved that PER is a state variable and its temporal perturbation is responsible for phase shifts induced by different LD cycles. Temporal PER2 induction by water pulses produced permanent phase shifts in behaviour in a phase-specific manner as light pulses did. This phase shifting mechanism has been suggested by numerous observations^30^,^31^,^34^,^35^,^36^ but has not been proven directly as with our approach. BMAL1 cannot be a state variable in a synthetic clock because it is too stable to generate 24-h oscillations (Fig. 1f) and previous genetic studies showed that oscillations and levels of BMAL1 are not critical for the clock^37^,^38^,^39^. By design principle and experimental demonstration, periods of synthetic oscillators are greatly affected by half-life of key components^19^. *Bmal1* mutant mice can be rescued by transgenic *Bmal1* controlled by either an endogenous or a constitutive promoter, but with little period difference^38^,^40^. In addition, constitutive overexpression of BMAL1 does not disrupt the clock significantly^39^. Similarly, the clock is insensitive to levels of CRY as constitutive overexpression neither disrupted the clock nor modulated period (Supplementary Fig. 7a). These data suggest that CRY is not limiting relative to PER, and synthetic oscillations of CRY may not affect endogenous PER oscillations and thus cannot produce tunable oscillations (Supplementary Fig. 7b). Our mathematical model predicts that tCRY1 would show low amplitude rhythms relative to endogenous PER rhythms, whether it is 24- or 30-h entrainment, due to its much longer half-life than that of PER. Since PER would still be limiting in these conditions, the phase and period of PER:CRY would still be determined by PER rhythms. An important design principle was revealed from our present study. Even though our inducible promoter is constitutive in nature, our DTG/DKO mice exhibited robust rhythms in *tPer* mRNA, protein and behaviour on *ad libitum* of Tet water. This was possible because circadian rhythms generated by the artificial clock and subsequent oscillations of the artificial clock reinforce and sustain each other due to their functional interdependence. Although the artificial oscillator may play a dominant role in setting properties of the oscillator, other endogenous clock genes certainly contribute to the parameters. When DTG/DKO or DTG/HKO mice are on Tet *ad libitum*, these mice take an endogenous period, ∼24 h, suggesting that the default period is affected by other endogenous clock genes. On the basis of numerous previous studies showing the importance of *Per* gene regulation at the transcriptional and epigenetic levels^41^,^42^,^43^,^44^, our results are surprising in that tight feedback regulation of CLOCK:BMAL1 on *Per* expression is dispensable for generating rhythms. Our data demonstrated that non-native and asymmetric oscillations of PER can sustain circadian rhythms suggesting that essential features of the circadian oscillator are encoded in the amino-acid sequence rather than the promoter of *Per* genes. Using these unique amino-acid signatures, such as the circadian degron in PER, it should be possible to generate diverse circadian switches or oscillators by employing the existing genetic network. For instance, a circadian transcription switch could be generated by fusing PER2 with a transcriptional activator or inhibitor in a brain-specific manner as in our experiments. As with PER2-Luciferase, which exhibits circadian oscillations in luciferase levels and activity^45^, the fusion proteins would oscillate in a circadian manner and should be tunable if the transgene is under drug regulation (Fig. 7). These design principles can be applied to any synthetic oscillators or switches that can control behaviour and physiology *in vivo*.

## Methods

### Mice

All mice were maintained in a climate-controlled room and used according to the Florida State University Animal Care and Use Committee's guidelines. All experiments involving animals were performed according to Committee-approved protocols. The double transgenic Scg2-TA; tetO-Per2 mice are on a C57BL/6 background (Jackson Laboratory #008284 (Scg2-TA), #01674 (tetO-Per2)) and were described previously^21^. WT C57BL/6 (Jackson Laboratory #000664) and ROSA26-rtTA transgenic (Jackson Laboratory #006965) mice were purchased from The Jackson Laboratory. The *mPer1/2* mutant mice were described previously^22^ and are available from the Jackson Laboratory (#010491 and #010492). The mutant mice were backcrossed with C57BL/6 mice for more than seven generations. Two- to 6-month-old male and female mice were used. *Per2*^*luc*^ knockin allele has been described previously^45^ and the knockin mice are available from the Jackson Laboratory (#006852). Briefly, a luciferase reporter was knocked into the endogenous *Per2* gene, between the last amino acid and the stop codon to produce a fusion transcript and protein with reporter capability in real time. The knockin did not disrupt any clock function of the *Per2* gene. *Rosa-rtTA; tetO-Per2; Per2*^*luc*^ mice were generated by crossing *Rosa-rtTA; tetO-Per2* with *Per2*^*luc*^ mice.

### Cells and antibodies

MEFs were prepared from embryos isolated from a pregnant female mouse at day-13 post coitum (assuming day one is the first day the plug is observed). The embryos were finely minced and then treated with 0.25 trypsin while being incubated at 37 °C for 30 min. The dissociated primary MEFs were grown and maintained in DMEM supplemented with 10% foetal bovine serum.

Antibodies to clock proteins were generated against recombinant clock proteins expressed in bacteria and thoroughly characterized using tissue extracts prepared from corresponding knockout mice by Lee *et al.*^27^,^46^ The sensitivity of the antibodies for detecting endogenous levels of each clock protein was confirmed by absence of the expected protein band in tissue extracts prepared from the corresponding knockout mice, while the band is present in control tissue extracts prepared from WT mice. PER1-1-R, PER2-1-R, CLK-1-GP, BM1-2-GP, C1-GP and C2-GP antibodies were used at 1:1,000 dilution in 5% milk–Tris-buffered saline containing 0.05% Tween 20 solution. These antibodies are readily available upon request. Rabbit anti-ACTIN antibody was purchased from Sigma (A5060) and used at 1:2,000.

### Adenoviral constructs and virus production

The construction of the recombinant adenoviral vectors encoding *tetO-Per1* and *CMV-rtTA* followed the procedure of He *et al.*^47^ Briefly, for the *tetO-Per1* and *tetO-Cry1* adenoviral vectors, TRE (tetracycline responsive element) from the pTRE2 plasmid (Clontech) was cloned into promoterless pAdTrack (a shuttle vector) along with a full-length *Per1* cDNA and a full-length *Cry1* cDNA, respectively. For *CMV-rtTA*, the cDNA coding for *rtTA* from pTet-On (Clonetech) was cloned into the pAdTrack-CMV shuttle vector. These shuttle vectors were subsequently cut with PmeI for linearization and then transformed into the *E.coli* BJ5183 strain along with the pAdEasy adenoviral backbone vector to generate complete adenoviral vectors by *in vivo* recombination. Generation and purification of recombinant adenovirus in packaging cells (HEK293) was also performed as described by He *et al.*^47^ Proper titres to achieve >95% infection efficiency were assessed by counting green fluorescent protein-expressing cells in culture plates infected with different concentrations of adenoviruses^21^.

### Measuring bioluminescence rhythms and clock proteins

MEFs were maintained in DMEM supplemented with 10% foetal bovine serum. To measure bioluminescence rhythms, MEFs were plated ∼90% confluent in 24-well plates the day before experiments. On the following day, different amounts of drugs were added into the wells of the plates after a 2-h serum shock with 50% horse serum in DMEM. For Supplementary Figs 2b and 7, the cells were infected with *tetO-Per1*- or *tetO-Cry1* and *rtTA*-adenovirus 24 h before drugs were added. These plates were sealed with cellophane tape and placed into a Lumicycle luminometer (Actimetrics, Wilmette, IL). For immunoblots with MEFs, cells were seeded in 60-mm dishes at 90% confluency 1 day before experiments. The cells were treated with drugs and collected 24 h later. For cycloheximide treatment, 40 μg ml^−1^ was added to cells 24 h after serum shock and cells were collected at indicated times after the treatment. For bioluminescence experiments, the results were reproduced in at least two independent experiments.

### Analysis of circadian behavioural rhythms

All mice were individually housed in wheel-running cages with free access to food and water. The animals were placed in a LD cycle (12:12) for at least 7 days, followed by constant darkness (DD) for at least 2 weeks to measure baseline activity. For *ad libitum* drug treatment, the water was replaced with water containing 50 μg ml^−1^ Tet (Fig. 2a–c) or 10 μg ml^−1^ doxycycline hydrochloride (Dox; Fig. 2b) plus 5% sucrose. Rhythmicity could be induced in all of the DTG/DKO and DTG/HKO mice (except for one mouse) anytime during DD by 50 μg ml^−1^ Tet- and regular water bottle changes, as shown in Fig. 5. Tet or Dox treatment did not affect rhythms in WT and *Per* mutant mice significantly (shown in the current study and previously^21^). A recent study suggested that administration of Tet and Dox at high doses could affect behavioural rhythms through toxicity on mitochondria, but the doses we used are at least 1–2 orders of magnitude lower than those that were shown to induce the toxicity^48^.

Wheel-running activity was recorded and analysed using ClockLab (Actimetrics, Wilmette, IL). Free-running period and relative power in the circadian range were calculated from the first 2 weeks of rhythmic period; for DTG/DKO and DTG/HKO mice in Fig. 5a, this corresponded to the first 2 weeks during the first water/Tet entrainment. Free-running period was calculated using a χ^2^-periodogram with 6-min resolution between hours 10 and 36 (ClockLab). The relative power (amplitude) of the circadian spectrum from 18–30 h was determined from a normalized Fast Fourier transform using a Blackman-Harris window (ClockLab). The period and power values were compared between two groups (for example, WT mice and mice that were DTG in a *Per* mutant background) and *P* values calculated using Student's *t*-test. Asterisks indicate significant *P* values as follows: *P<0.05; **P<0.01; ***P<0.001. Data across multiple experiments are shown as mean+/−s.e.m.. The quantitative analysis of phase shift in Fig. 5d were calculated using changes in stable phase before and after light or water pulses^49^. The stable phases were determined using linear regression on the activity onset points for 7 days. The phase shifts were measured as differences (in hours) between activity onset times before and after the treatment.

For 30-h entrainment, mice were entrained in 24-h LD, DD for 2 weeks and then given Tet *ad libitum* for 24 h followed by 6 h of regular water every 30 h while in DD. As with 24-h entrainment (for example, Fig. 4a, 6 h of regular water every 24 h), there were at least several days of transitional period between two different schedules of 30-h entrainment (Supplementary Fig 8). The χ^2^-graphs in Fig. 5c were generated from the same period as shown in the 30-h actograms.

### Tissue collection immunoblots and quantitative RT–PCR

Liver and brain tissues were collected from WT mice on the second day in DD. The tissues from DTG/DKO mice were collected when they were rhythmic through receiving Tet *ad libitum* for at least a week. DKO mice were never rhythmic by the same treatment but tissues were collected at the same time. Immunoblotting was performed as follows. Tissues were homogenized at 4 °C in three volumes of extraction buffer (0.4 M NaCl, 20 mM HEPES (pH 7.5), 1 mM EDTA, 5 mM NaF, 1 mM dithiothreitol, 0.3% Triton X-100, 5% glycerol, 0.25 mM phenylmethylsulfonyl fluoride, 10 mg of aprotinin per ml, 5 mg of leupeptin per ml, 1 mg of pepstatin A per ml). Homogenates were cleared by centrifugation (twice, 12 min each, 12,000 *g*). Supernatants were mixed with 2 × sample buffer and boiled. Proteins were separated by electrophoresis through SDS polyacrylamide gels and then transferred to nitrocellulose membranes. Membranes were blocked with 5% non-fat dry milk in Tris-buffered saline containing 0.05% Tween 20 and then incubated with primary antibodies overnight followed by incubation with secondary antibodies for 1 h. The blots were developed using an Enhanced Chemiluminescence substrate (WestFemto, ThermoFisher Scientific, Waltham, MA). RT–PCR was performed using the following primers.

*Per1* sense 5′-TCCCTGTTTCGTCCTCCACT-3′, antisense 5′-CTTGAGCCATTGCTGTTTGC-3′, *Per2* sense 5′-ATGAATGGATACGTGGACTTCTCCCCA-3′, antisense 5′-CAGGGTTGCCAGCGTGCTGGCCTT-3′, β*-Actin* sense 5′-ATG GGTCAGAAGGACTCCTATGTGGG-3′, antisense 5′-GGCCACACGCAGCTCATTGTAGAAGG-3′.

Immunoblots shown in Figs 1b,f and 3b and Supplementary Fig. 1 were reproduced in at least three experiments. The original scanned images of the blots shown in Figs 1 and 3 are shown in Supplementary Fig. 9.

### Mathematical model

To study the synthetic oscillations in DTG/DKO and DTG/HKO mice driven by Tet or Dox oscillations, we have modified the detailed mathematical model of the mammalian circadian clock developed by Kim and Forger^12^, which has been successfully used to study pharmacological manipulation of circadian rhythms and the effects of introducing a new circadian clock component^50^,^51^. In the modified model, the following equations were added to simulate Tet or Dox rhythms driven by rhythmic water intake.


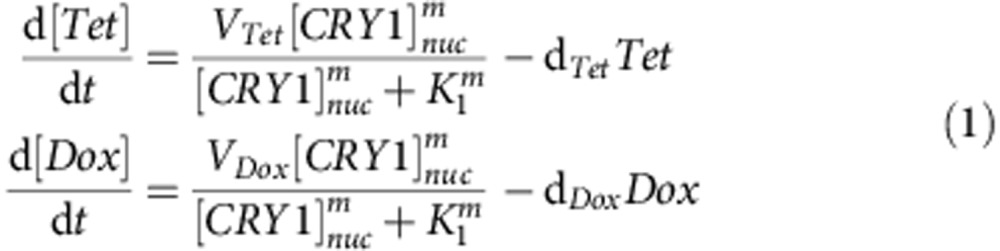


Because the water drinking mainly occurs during the active phase (CT12 to CT24)^52^, we assumed that the phase of water-intake rhythms follows the phase of CRY protein in the nucleus, whose peak occurs around ZT 18 (midway between lights-off and lights-on in a 12:12 LD cycle)^27^. We assumed that the half-life of Tet and Dox are 2 and 6 h, respectively, matching experimental data^53^,^54^. To simulate the 6-h water pulse, we used a modified water-intake rate


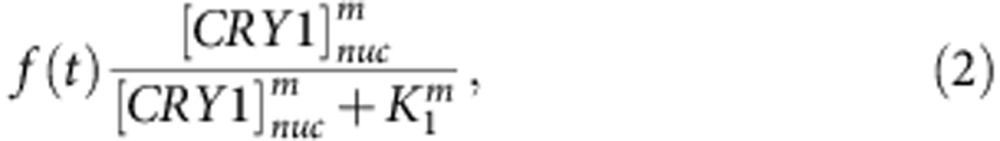


where


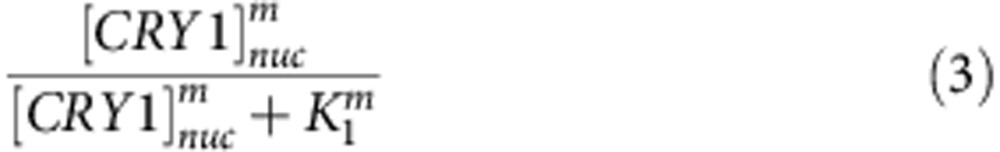


is the original water-intake rhythms in *ad libitum* conditions in the model. *f*(*t*) is the periodic function that has the period of water-pulse rhythms (24 or 30 h) and has the value of 1 during the 6-h pulse and 0 for the rest of the period.

To simulate the repression of *tPer* transcription by Tet/Dox in the Tet-Off system, the following equation was used.


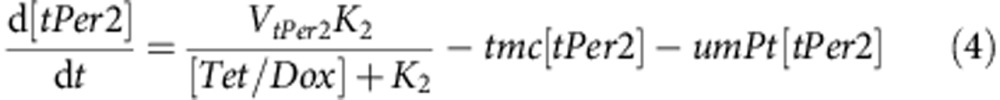


In the model, we assumed that *tPer2* transcripts behave the same as the endogenous *Per2*, and thus we adopted the same parameters for the nuclear export rate (*tmc*) and the degradation rate (*umPt*) as those of endogenous *Per2*. We also used the same parameter for posttranslational regulation of tPER2 as those of endogenous PER2. Supplementary Table 1 summarizes newly added and modified parameters to the original model described in Kim and Forger^12^.

To simulate constitutive expression of tPER2 in Fig. 1e, we used following equation:


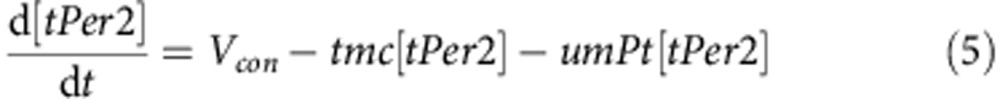


where *V*_*con*_=0, 3.25, 13 and 26 nM h^−1^ for zero, one-, four- and eightfold expression, respectively in Fig. 1e because the average level of endogenous PER2 is 3.25 nM h^−1^r in the model.

All the simulations were performed using Mathematica 10.0 (Wolfram Research).

## Additional information

**How to cite this article:** D'Alessandro, M. *et al.* A tunable artificial circadian clock in clock-defective mice. *Nat. Commun.* 6:8587 doi: 10.1038/ncomms9587 (2015).

## Supplementary Material

Supplementary Information Supplementary Figures 1-9, Supplementary Table 1 and Supplementary References.
