# Supplementary material for: A tunable artificial circadian clock in clock-defective mice
Source: Nat Commun. 2015 Nov 30;6:8587. doi: 10.1038/ncomms9587 (PMC4674671; doi:10.1038/ncomms9587)
Supplement: Supplementary Information — Supplementary Figures 1-9, Supplementary Table 1 and Supplementary References. [file ncomms9587-s1.pdf]

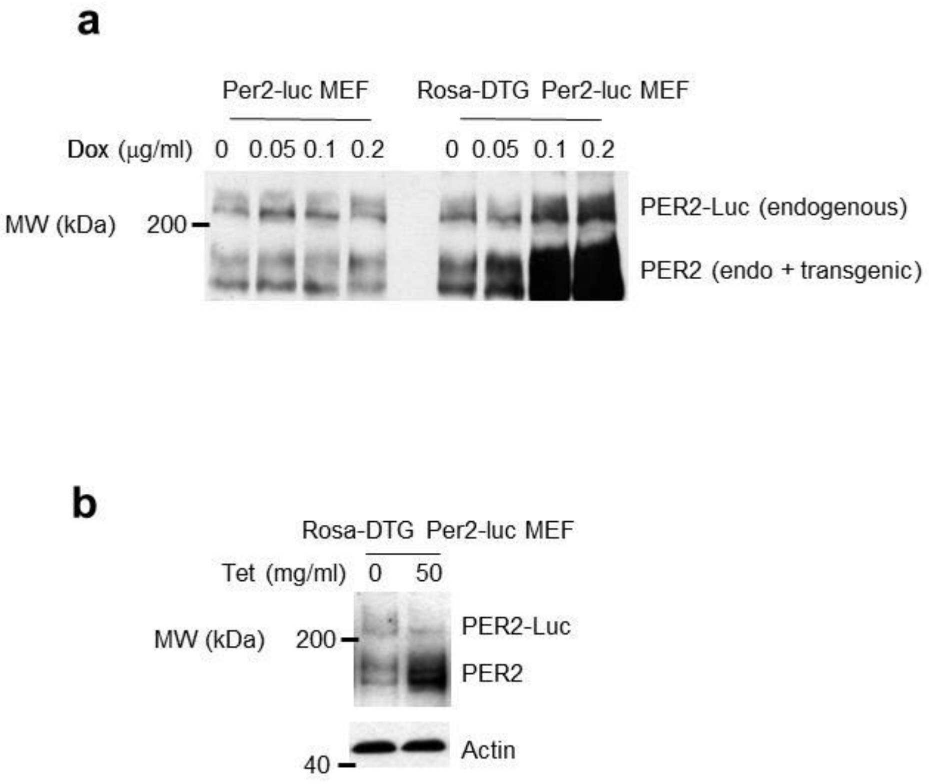

Supplementary Figure 1. Expression of the inducible tPER2 is proportional to Dox/Tet concentration in *Rosa-DTG/Per2* $^{Per2\text{-}luc/wt}$ MEFs. **(a)** Dose-responsive expression of tPER2 by Dox. Note that there are three PER2 isotypes: endogenous PER2, endogenous PER2-LUC and transgenic PER2. PER2-Luc is used for measuring bioluminescence rhythms. There were low basal levels of tPER2 in the absence of Dox. (**b)** Expression of tPER2 by Tet. Expression levels of tPER2 induced by Tet (50-200 µg/ml) were lower than those induced by Dox but were still close to endogenous levels. tPER2 has exactly the same amino acid sequence as endogenous PER2[1]. These immunoblots were reproduced in at least three experiments.

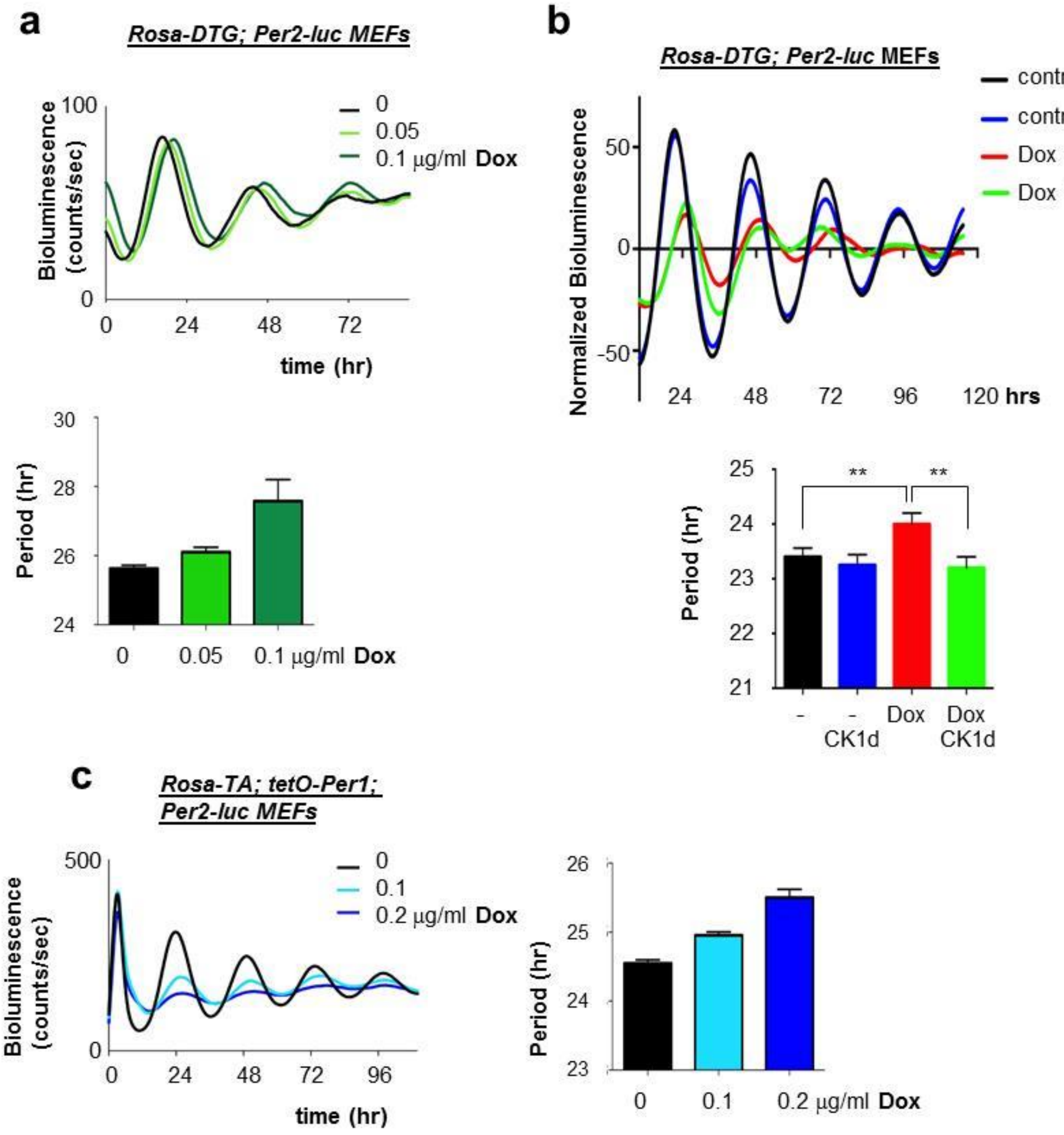

Supplementary Figure 2. Period is lengthened by Dox in a dose-responsive manner. **(a)** Inducible *Per2* in *Per2-luc* cells. Different amounts of Dox were added after a 2-hr serum shock. n=4 for each dose level. Values are mean+/-SEM. **(b)** Rescue of period lengthening in tPER2-overexpressed cells by transgenic expression of CK1δ. Extra amounts of CK1δ by the transgenic expression in control cells had little effect on period because endogenous CK1δ/ε are already in excess to PER. However, the transgenic expression of CK1δ rescued period lengthening in tPER2-expressing cells by restoring stoichiometry of PER to CK1δ/ε. n=4 for each group. Values are mean+/-SEM. *p<0.05, **p<0.01, ***p<0.001 (unpaired, two-tailed *t*-test). **(c)** Inducible *Per1* in *Per2-luc* cells. Period and amplitude are altered in a similar manner by Dox in *Rosa-rtTA;tetO-Per1; Per2* $^{Per2\text{-}luc/wt}$ MEFs. Rosa-*Per1*-DTG cells were generated by transducing adenoviral *Rosa-rtTA* and *tetO-Per1* into *Per2-luc* MEFs. n=4 for each dose level. Values are mean+/-SEM. Representative of two experiments.

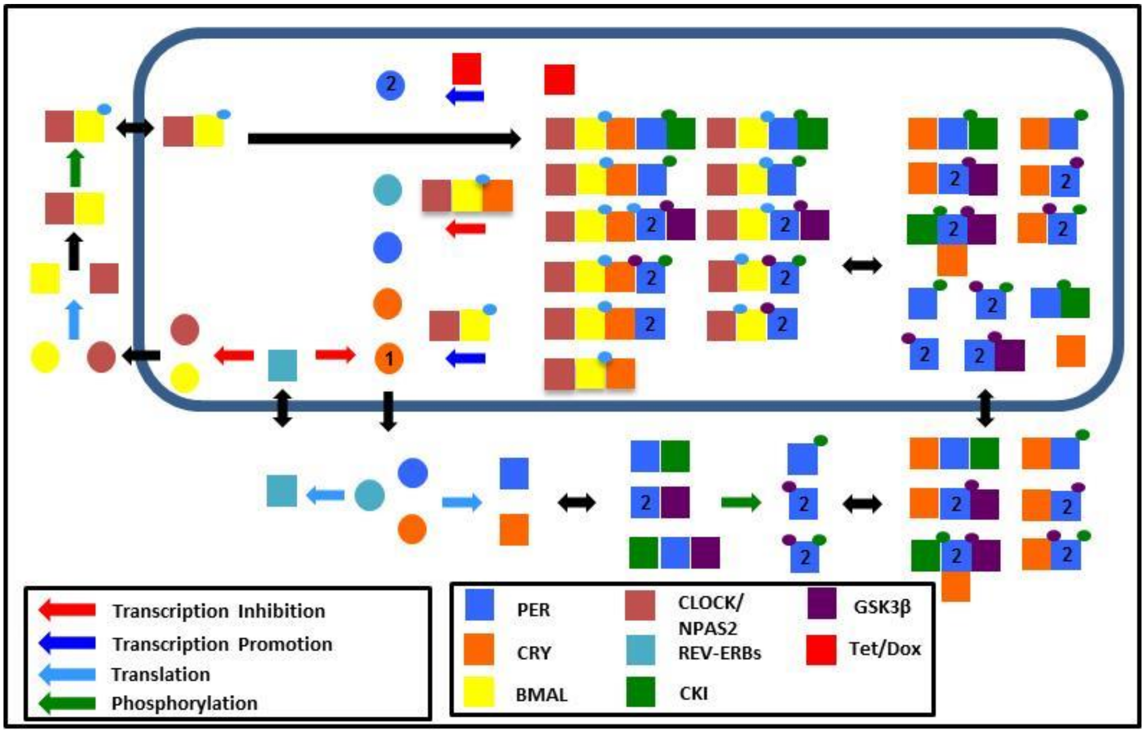

Supplementary Figure 3. Schematic of a mammalian circadian clock model with the Tet-controlled *tPer2* system. This model is adapted and modified from studies of Kim and Forger[2]. In this model, Tet/Dox promotes transcription of *tPer2* (Tet-ON) (top part of the diagram) or represses it (Tet-OFF). Circles and squares refer to transcripts and proteins, respectively. Numbers denote different family members. Small circles refer to phosphorylation mediated by the same color-coded kinases. This model was originally developed to simulate phenotypes of clock gene knockout and knockdown mutants and incorporate accurate stoichiometric relationships among clock proteins[2]. See the methods for details of this model such as equations and parameters.

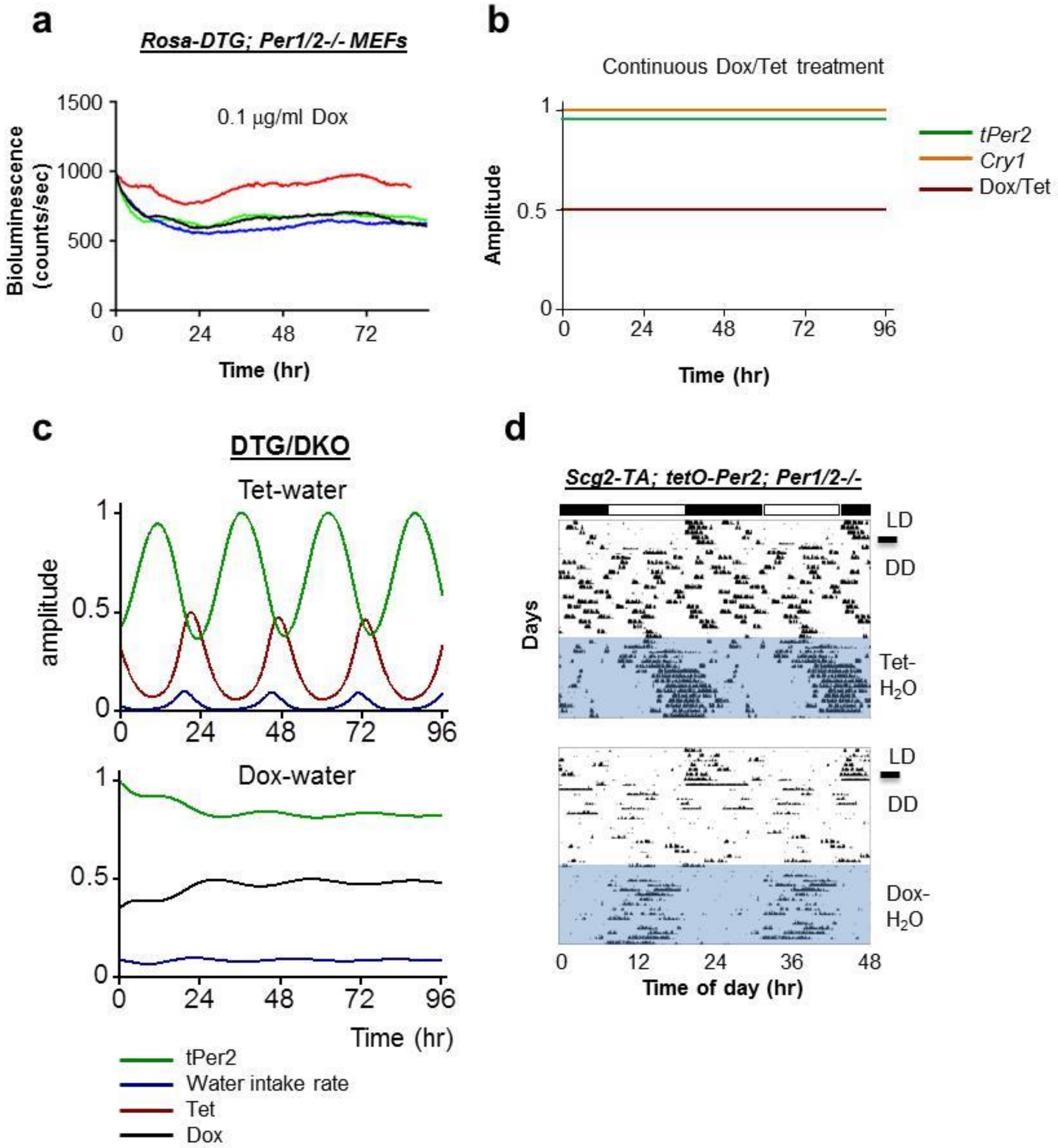

Supplementary Figure 4. Oscillations in Dox or Tet are required for generation of *tPer2* mRNA and other circadian rhythms. **(a)** Bioluminescence rhythms from *Per2* promoter-luciferase[1] were measured in the presence of Dox in Rosa-DTG/DKO MEFs. 4 traces are shown (n=4). **(b)** The results were simulated by our mathematical model assuming that Tet or Dox levels do not fluctuate. **(c)** Circadian drinking behavior can generate rhythms in Tet and *tPer2* mRNA in vivo. A mathematical model predicts that circadian drinking behavior can generate sustained rhythms of *tPer2* mRNA with Tet, but not with Dox in DTG/DKO. The model assumes that drinking mainly occurs during the active phase (CT12 to CT24)[3]. The half-lives of Tet and Dox were assumed to be 2 hours and 6 hours, respectively[4, 5]. Due to the long half-life of Dox, circadian Dox rhythms were weak or could not be sustained. Water intake rate (blue line) is described as equation (3) in the model (see the Methods for details). **(d)** Similar results were obtained in vivo when DTG/DKO mice were on Dox or Tet ad libitum. n=4 each. Representative actograms are shown.

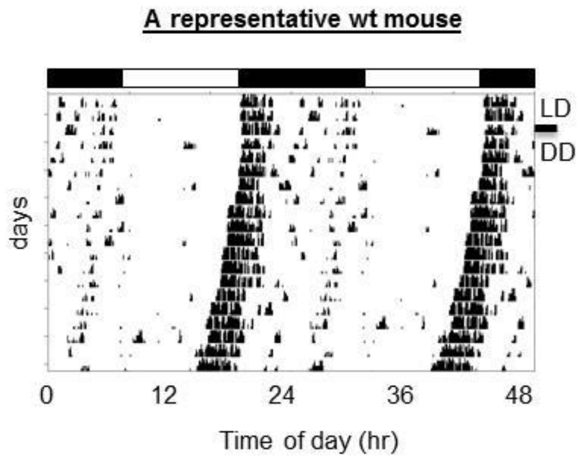

Supplementary Figure 5. A representative actogram for wt mice. This is one of 8 wt mice used to calculate amplitude and period in Fig. 2 and 5.

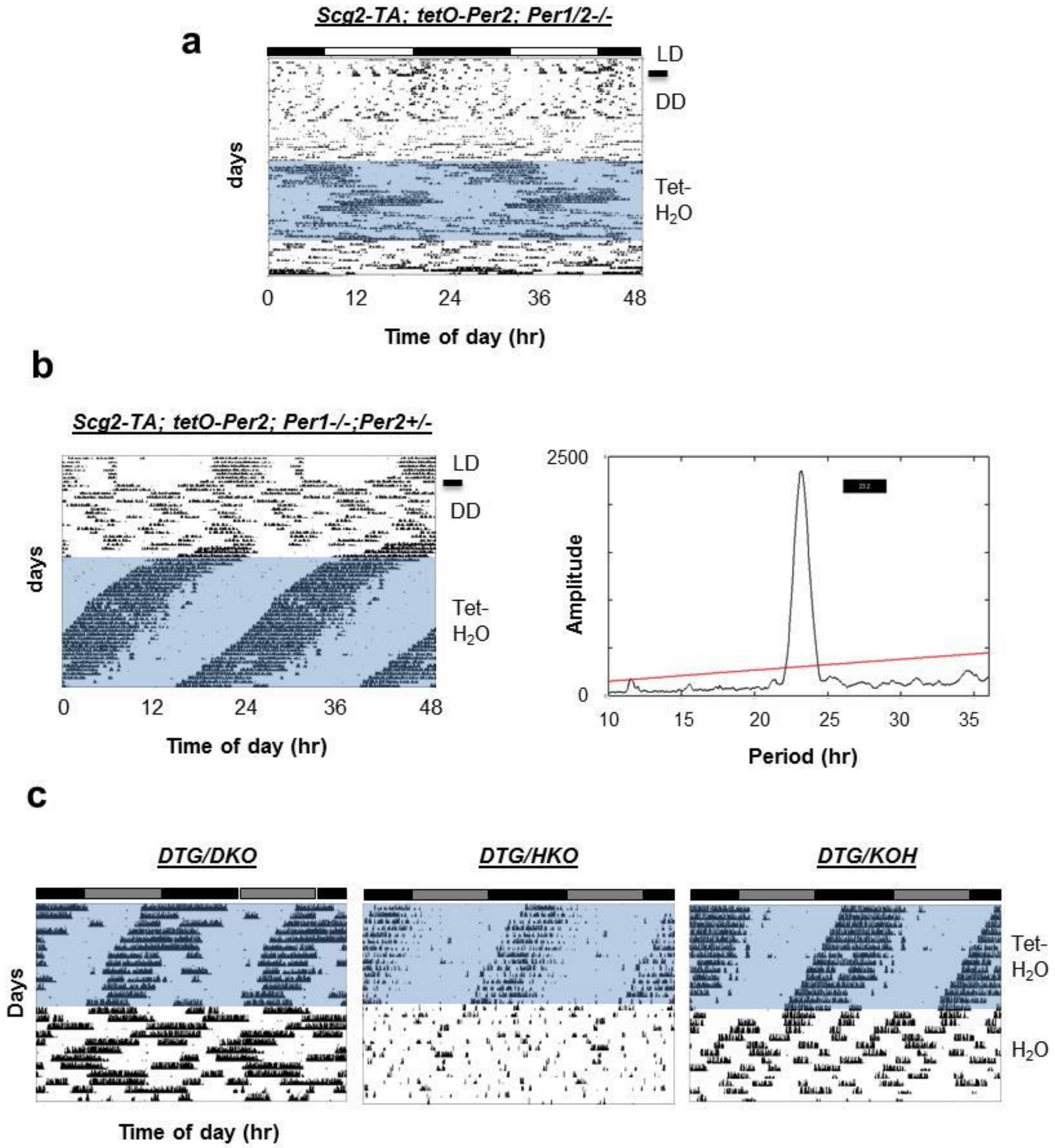

Supplementary Figure 6. DTG/DKO and DTG/KOH mice are rhythmic when they are on Tet ad libitum. **(a)** Some DTG/DKO mice on Tet ad libitum (3 out of 7) lost rhythmicity gradually. **(b)** DTG/KOH mice show robust amplitude rhythms as wt mice. Representative of 3 mice. **(c)** Induced rhythms disappear almost immediately when Tet water is changed to regular water. n=3 each.

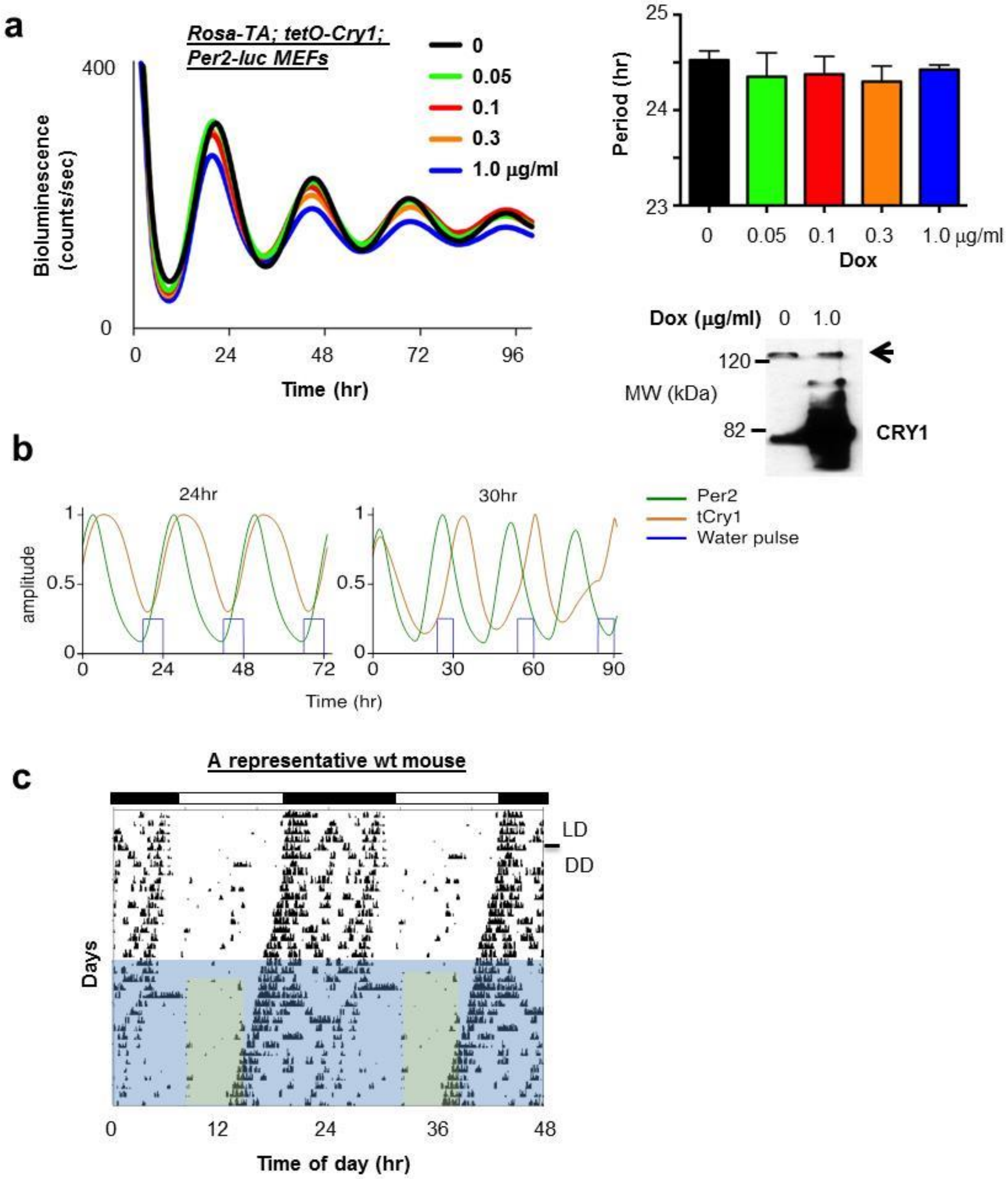

Supplementary Figure 7. CRY cannot be used for an artificial oscillator. **(a)** CRY1 overexpression in *Rosa-rtTA;tetO-Cry1; Per2* $^{Per2\text{-}luc/wt}$ MEFs did not affect circadian rhythms. CRY1 was overexpressed by different doses of Dox in the cells. Period is shown as mean+/-SEM, n=4. p>0.05 (unpaired, two-tailed *t*-test). CRY1 was significantly overexpressed relative to endogenous CRY1, which is shown in the immunoblot. The arrow indicates a nonspecific band. **(b)** Our mathematical model also predicted that PER rhythms and possibly behavioral rhythms would exhibit ~24 hr period even if *Cry1*rhythms are entrained to a 30 hr Tet-water cycle in Cry-deficient mice. *tCry1* was entrained to 24 and 30 hr cycles in *Cry*-deficient mice *in silico*. The results represent the final several days of more than 20 days of simulation under 24- and 30-hr entrainment. **(c)** Scheduled water treatment does not affect circadian rhythms wt mice. Representative of 3 mice.

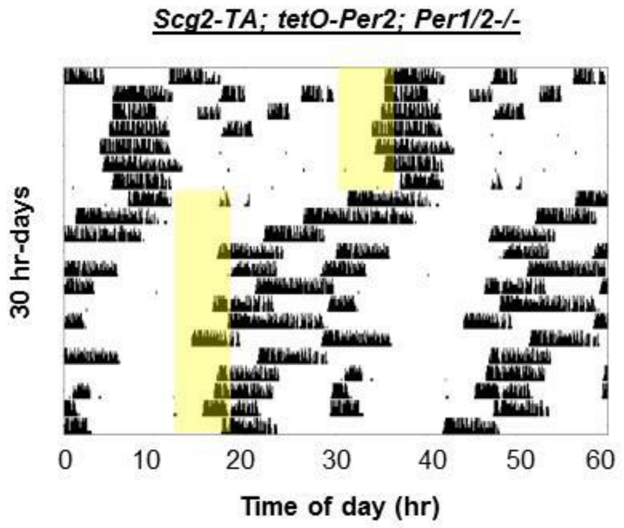

Supplementary Figure 8. There are several days of transitional period between two different schedules of 30-hour entrainment. Representative of 3 mice. The mice were on Tet water continuously except for 6 hrs in every 30 hrs, as in Fig. 6. The mice were subjected to two different schedules of 30-hour entrainment as indicated by two yellow areas.

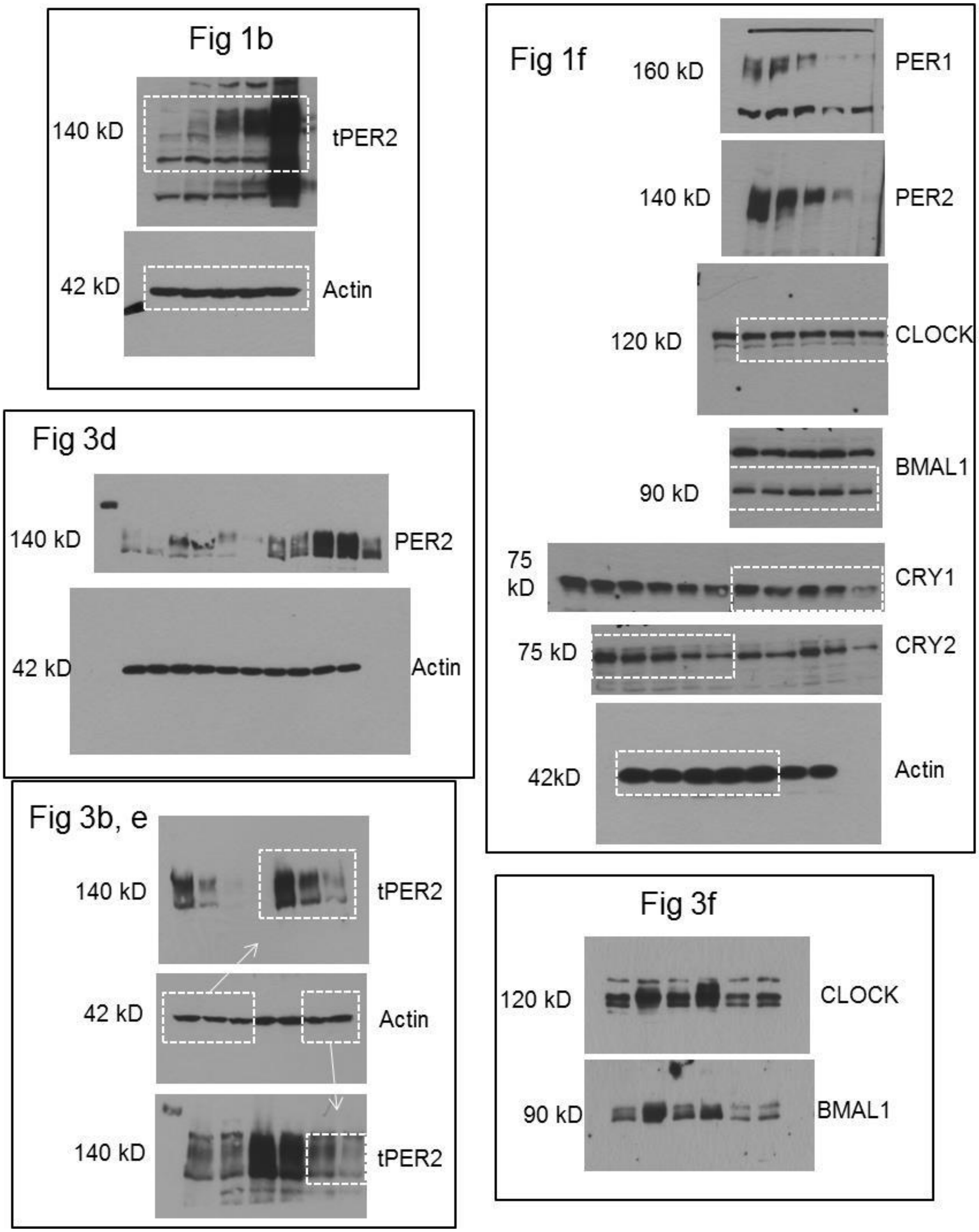

Supplementary Figure 9. The original scanned images of the blots shown in Fig. 1 and 3.

Supplementary Table 1. Newly added and modified parameters to the Kim and Forger mathematical model.

Parameter Description	Symbol	Value
Maximal production rate constant for Tet with water (50 μg/ml Tet) consumption	$\mathbf{V_{Tet}}$	110nM/hr
Half-maximal constant for Tet or Dox production	$\mathbf{K_1}$	9.8nM
Hill-coefficient for Tet or Dox production	**m**	5
Degradation rate constant for Tet	$\mathbf{d_{Tet}}$	Log2/2/hr
Maximal production rate constant for Dox with water (10μg/ml Dox) consumption	$\mathbf{V_{Dox}}$	15nM/hr
Degradation rate constant for Dox	$\mathbf{d_{Dox}}$	Log2/6/hr
Maximal transcription rate constant for *tPer2* via Tet or Dox	$\mathbf{V_{tPer2}}$	17.5nM/hr
Half-maximal concentration of Tet or Dox for *tPer2* transcription	$\mathbf{K_2}$	16nM
Nucleus export rate constant for *tPer2* mRNA	tmc	0.16426/nr
Degradation rate constant for *tPer2* mRNA	umPt	0.58892/hr
Transcription rate constant for *Per1* mRNA*	trPo	25.92nM/hr
Binding rate constant for REV-ERBs to GSK3β*	ag	0/nM hr
Binding rate constant for PER2 to GSK3β*	agp	0.27924 /nM hr
Degradation rate constant for *Rev-Erb*s mRNA*	umRev	0.7551/hr
Degradation rate constant for REV-ERBs*	uRev	0.8244/hr

8 new parameters are highlighted in bold and 5 modified parameters are marked with '*'. See Supplementary Table 3 in Kim and Forger [2] for a complete list of parameters.

Supplementary References

1. Chen, R. *et al.* Rhythmic PER abundance defines a critical nodal point for negative feedback within the circadian clock mechanism. *Molecular cell* **36**, 417-430 (2009).
2. Kim, J.K. & Forger, D.B. A mechanism for robust circadian timekeeping via stoichiometric balance. *Molecular systems biology* **8**, 630 (2012).
3. Noh, J.Y. *et al.* Presence of multiple peripheral circadian oscillators in the tissues controlling voiding function in mice. *Experimental & molecular medicine* **46**, e81 (2014).
4. English, A.R. & Lynch, J.E. Alpha-6-deoxyoxytetracycline. II. Activity in chemotherapeutic studies in the mouse. *Proc Soc Exp Biol Med* **124**, 586-591 (1967).
5. Schonig, K. & Bujard, H. Generating conditional mouse mutants via tetracycline-controlled gene expression, in *Transgenic Mouse Methods and Protocols*, Vol. 209. (eds. M.H. Hofker & J. van Deursen) 69-104 (Humana Press, New Jersey; 2002).
